# Estimation of Optimal and Maximum Standardized Ileal Digestible Methionine Requirements Based on Performance for Male Broilers Aged 0 to 21 Days

**DOI:** 10.3390/ani15020278

**Published:** 2025-01-20

**Authors:** Su-Hyun An, Changsu Kong

**Affiliations:** 1 Research Institute for Innovative Animal, Kyungpook National University, Sangju 37224, Republic of Korea; woobi89@gmail.com; 2 Department of Animal Sciences, The Ohio State University, Columbus, OH 43210, USA; 3 Department of Animal Science and Biotechnology, Kyungpook National University, Sangju 37224, Republic of Korea; 4 Department of Animal Science, Kyungpook National University, Sangju 37224, Republic of Korea

**Keywords:** amino acid requirement, methionine, standardized ileal digestible, broiler

## Abstract

Two experiments were conducted with Ross 308 male broiler chickens to determine the standardized ileal digestible (SID) methionine (Met) requirement for maximum weight gain during the starter (0 to 10 days) and grower (10 to 21 days) phases. A total of 720 and 288 birds were allocated to Experiments 1 and 2, respectively, and assigned six dietary treatments in a randomized complete block design. The experimental diets were formulated with corn, soybean meal, and synthetic amino acids to achieve varying SID Met levels (3.8 to 5.8 g/kg in experiment 1 and 3.0 to 5.5 g/kg in experiment 2), with increments of 0.4 and 0.5 g/kg. Weight gain, feed intake, and the gain-to-feed ratio (G:F) increased quadratically (*p* < 0.05) as dietary SID Met increased. The SID Met requirements for maximum weight gain and G:F in the starter phase were 5.1 g/kg (79.5 mg/d) and 5.3 g/kg (89.8 mg/d), while in the grower phase, they were 5.5 g/kg (343.0 mg/d) and 5.4 g/kg (330.3 mg/d), respectively. These findings, divided into two growth phases, provide guidelines to facilitate the optimal and maximum performance of young male broilers, and this may facilitate the selection of appropriate values for formulating broiler feed. These findings also highlight the importance of Met supplementation strategies for improving performance.

## Simple Summary

Methionine (Met) is widely recognized as the first limiting amino acid in broiler chickens fed diets based on corn and soybean meal, and it plays a critical role in various biological functions. The metabolism of Met is closely associated with cysteine (Cys), indicating that Cys levels may impact Met requirements. Ensuring optimal dietary concentrations of Met is linked to enhanced feed efficiency, which, in turn, promotes growth and overall performance in broiler chickens. Therefore, the present study aimed to determine the dietary Met requirements based on the growth stage of the birds and the levels of dietary Met and Cys. Two experiments involving Ross 308 broiler chickens were conducted to identify the optimal standardized ileal digestible (SID) Met levels for growth. During the starter phase (0 to 10 days), the optimal SID Met levels for maximizing weight gain and feed efficiency were found to be 5.1 g/kg (79.5 mg/d) and 5.3 g/kg (89.8 mg/d), respectively. In the subsequent phase (10 to 21 days), the ideal SID Met levels were 5.5 g/kg (343,0 mg/d) and 5.4 g/kg (330.3 mg/d).

## 1. Introduction

Methionine (Met) is the most limiting amino acid (AA) in broilers fed corn–soybean meal (SBM) diets [1]. It plays a crucial role in methyl-group metabolism and various biological functions, including maintaining normal protein metabolism and breast meat yield in birds [2,3,4]. Adequate dietary Met supplementation is vital for optimal growth and productivity. Imbalances, whether due to a deficiency or excess of Met, can reduce dietary AA availability, leading to undigested and unabsorbed AAs being excreted, which interferes with the bird’s growth response [5]. Therefore, accurately estimating optimal dietary Met requirements is essential for supporting broiler growth potential and increasing poultry productivity. Optimal dietary Met intake ensures efficient utilization for proper growth and productivity in broilers [6].

Feed constitutes a significant portion of poultry production expenses, accounting for approximately 50 to 70% of total costs. Improving feed formulation is essential for enhancing feed efficiency and reducing expenses while achieving production goals. Optimal bird performance relies on providing precise nutrient quantities that maximize growth potential. Recent research focuses on estimating the exact Met requirement, which is crucial for meeting birds’ growth potential.

The dietary Met concentration for optimal growth varies across different growth stages [7,8,9,10]. As birds age, their recommended dietary Met levels decrease [11,12]. For broilers, Rostagno et al. [9] suggested optimal digestible total sulfur amino acid (TSAA) concentrations (g/kg) of 5.4 (d 1 to 7), 5.2 (d 8 to 21), 4.6 (d 22 to 33), and 4.2 (d 34 to 42), according to growth phase. The Ross 308 nutrition specifications [7,8] recommend digestible Met levels of 5.1 g/kg for the starter phase (d 0 to 10), 4.7 g/kg for the grower phase (d 11 to 24), and 4.5 g/kg for the finisher phase (d 25 to market age), targeting birds weighing less than 1.6 kg. Aviagen [13] recommends for digestible Met concentrations in birds under 2.0 kg body weight (BW) 5.5 g/kg for the starter phase, 5.1 g/kg for the grower phase, and 4.8 g/kg for the finisher phase. Additionally, studies were presented regarding the recommended Met levels for optimal performance, specifically expressed as the Met + Cys to Lys ratio [6,14]. Like other nutrients and AA, the optimal dietary Met concentration is influenced by the growth stages and growth potential of birds. As feed consumption increases with bird age, the dietary recommended nutrient concentrations decrease. Dietary Met significantly impacts bird growth performance. Adjusting dietary nutrient composition during early chick stages is crucial for enhancing early growth and overall broiler performance.

Because Met requirements for growth can vary according to both the age of the birds and the statistical methods employed, most previous studies have examined the 0–21-day period as a single phase or as a later phase [15,16,17,18]. In contrast, this research performs Met recommendations by examining two distinct intervals—0–10 days and 10–21 days. This approach may allow for more detailed nutritional guidelines to promote optimal early growth. Variations in statistical methods can also influence these requirements. Information from various factors, including age and statistical methodologies, is valuable for improving the accuracy of Met requirement determination. This study aimed to estimate the optimal dietary standardized ileal digestible (SID) Met requirements for male broilers aged 0 to 21 days using different regression models.

## 2. Materials and Methods

All protocols used in the study were approved by the Animal Care and Use Committee of Kyungpook National University (approval number: KNU 2017-0140 and 2019-0125).

### 2.1. Experimental Diets

All experimental diets in both experiments comprised corn–SBM-based mash diets with varying SID Met concentrations. Experiment 1 had dietary concentrations ranging from 3.8 to 5.8 g/kg (Table 1) and included graded levels of SID Met and cysteine (Cys) at a constant Met:Cys ratio of 41:59 (Table 2). In contrast, Experiment 2 had concentrations varying from 3.0 to 5.5 g/kg (Table 3), with the diets incorporating graded levels of SID Met and Cys at a constant Met:Cys ratio of 50:50 (Table 4). These diets were formulated using the ideal digestible AA ratios relative to lysine (Lys) for the starter (6 to 14 days of age) and grower (15 to 35 days of age) phases, as reported by Hoehler et al. [19]. The SID AA content in the corn and SBM used in the diets was calculated using the equations described by Kong and Adeola [20]. The SID AA values for corn and SBM for 10-day-old and 21-day-old male broilers were derived from a previous study [21]. All indispensable AA levels, except for Met, were set at 110% of the levels suggested by Hoehler et al. [19] to prevent deficiencies in AAs other than Met.

### 2.2. Animal and Experimental Management

A uniform procedure was followed throughout both experiments, which were conducted independently. Experiment 1 involved 720 day-old male broilers (Ross 308), while Experiment 2 used 288 10-day-old male broiler chickens. Each bird was individually tagged and weighed for identification. The birds were subsequently allocated to six treatments in a randomized complete block design, with eight and six replicate cages per treatment in Experiments 1 and 2, respectively. Furthermore, Experiments 1 and 2 had 15 and 8 birds per cage, respectively. Each cage served as an individual experimental unit. Throughout the experimental period, all birds were provided with experimental mash diets and had access to water ad libitum. The environment was carefully controlled with continuous lighting, ventilation, and temperature regulation with an auto-mechanical system. The ventilation system was adjusted to maintain relative humidity levels within the range of 40% to 60%, contingent upon the temperature of the facility. For the first three days of birds, the room temperature was maintained at 33 °C and then gradually reduced by 2 °C per week until it reached 26 °C by day 21, in accordance with the recommendations for Ross 308 broilers. Thereafter, the temperature was maintained at 26 °C until the end of the experiment.

### 2.3. Performance Measurements and Chemical Analysis

Daily mortality data were recorded for each cage throughout the experimental period. Additionally, individual body weights, feed supply, and leftovers per cage were recorded at the beginning and end of each experiment to calculate body weight gain (BWG), feed intake, and the gain-to-feed ratio (G:F). The dry matter and AA compositions of the experimental diets were subjected to chemical analysis. The samples were ground using a Cyclotec™ Mill (CT 293 Cyclotec; Foss, Denmark). Corn, SBM, commercial diet, and experimental diet samples were analyzed for AAs (method 994.12; method 988.15) following the methods established by the Association of Official Agricultural Chemists International [22].

### 2.4. Statistical Analysis

BWG and G:F data were analyzed using the MIXED procedure in SAS Version 9.4 (SAS Inst. Inc., Cary, NC, USA). Orthogonal polynomial contrasts were used to assess linear and quadratic trends across dietary SID Met concentrations. Both AIC (Akaike information criterion) and BIC (Bayesian information criterion) values can be used to compare linear and quadratic regression models, but corrected AIC (AICc) was chosen as the primary criterion due to its adjustment for small sample size bias. The SID Met requirements were estimated using nonlinear regression analysis in SAS, utilizing one-slope broken-line and quadratic models based on the approach described by Robbins et al. [23]. The experimental diets were treated as fixed effects in the analysis, and the blocks, determined by BW per cage, were treated as random effects. The experimental unit was a cage, and statistical significance was set at *p* < 0.05.

## 3. Results

Two experiments were conducted to determine the SID Met requirements for broilers during different feeding phases: starter (0–10 days of age) and grower (10–21 days of age). Mortality data were recorded throughout the experimental period, were 4.9% for experiment 1 and 0.7% for experiment 2, respectively, and the feed intake of deceased birds was calculated and subsequently excluded from the final dataset.

### 3.1. Growth Performance

Table 5 presents data on the growth performance of birds. Graded levels of dietary SID Met demonstrated quadratic effects (*p* < 0.05) on final BW, BWG, and feed intake, while the G:F exhibited a linear response (*p* < 0.01). In Experiment 2, broilers fed graded levels of SID Met showed significant (*p* < 0.01) quadratic effects on final BW, BWG, and feed intake (Table 6). The growth performance of the birds was influenced by the supplemented concentrations of SID Met. In addition, the AICc values obtained from quadratic models were lower than those of the linear model in both experiments, indicating that quadratic models are better at explaining the data compared to the linear model.

### 3.2. Methionine Requirement Estimates

**Figure 1:**
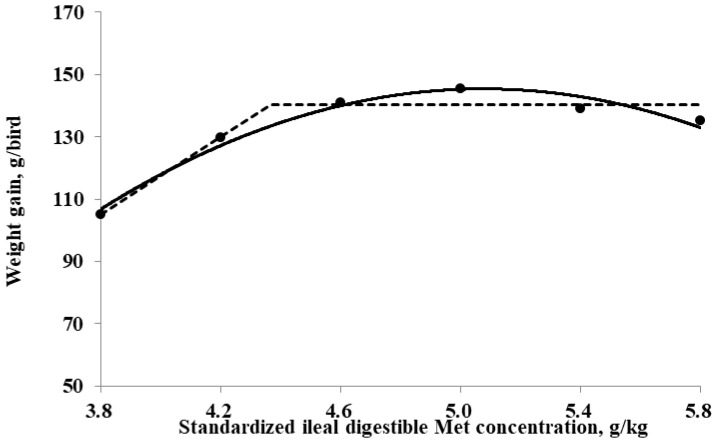
Fitted broken (dotted) and quadratic (solid) lines based on weight gain (g/bird) in 0–10-day-old broilers as a function of standardized ileal digestible (SID) Met in their diets (Experiment 1). Data points are expressed as the means of eight cages per treatment. The one-slope broken-line model indicates that the SID Met requirement was 4.4 g/kg (Y = 140.2 + 61.9 × [X − 4.4] [X < 4.4]; *p* = 0.01; R^2^ = 0.949; SE = 0.11; CI 95% = 4.0 to 4.7). The quadratic-line model shows that the SID Met requirement was 5.1 g/kg (Y = 145.5 − 23.7 × [5.1 − X] × [5.1 − X] [X < 5.1]; *p* = 0.01; R^2^ = 0.968; SE = 0.05; CI 95% = 4.98 to 5.17), and 95% of the upper asymptotic value of the quadratic model indicates that the SID Met requirement was 4.8 g/kg.

**Figure 2:**
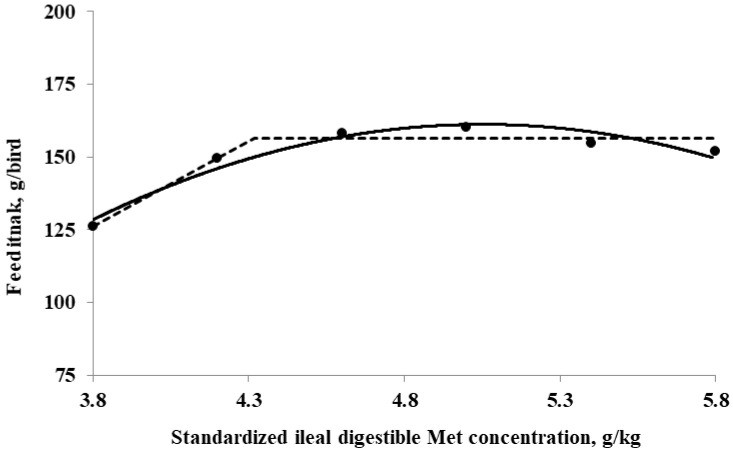
Fitted broken (dotted) and quadratic (solid) lines based on feed intake (g/bird) in 0–10-day-old broilers as a function of standardized ileal digestible (SID) Met in their diets (Experiment 1). Data points are expressed as the means of eight cages per treatment. The one-slope broken-line model indicates that the SID Met requirement was 4.3 g/kg (Y = 156.3 + 58.1 × [X − 4.3] [X < 4.3]; *p* = 0.01; R^2^ = 0.946; SE = 0.09; CI 95% = 4.0 to 4.6). The quadratic-line model shows that the SID Met requirement was 5.1 g/kg (Y = 161.2 − 20.9 × [5.1 − X] × [5.1 − X] [X < 5.1]; *p* = 0.01; R^2^ = 0.947; SE = 0.09; CI 95% = 4.87 to 5.23), and 95% of the upper asymptotic value of the quadratic model indicates that the SID Met requirement was 4.8 g/kg.

**Figure 3:**
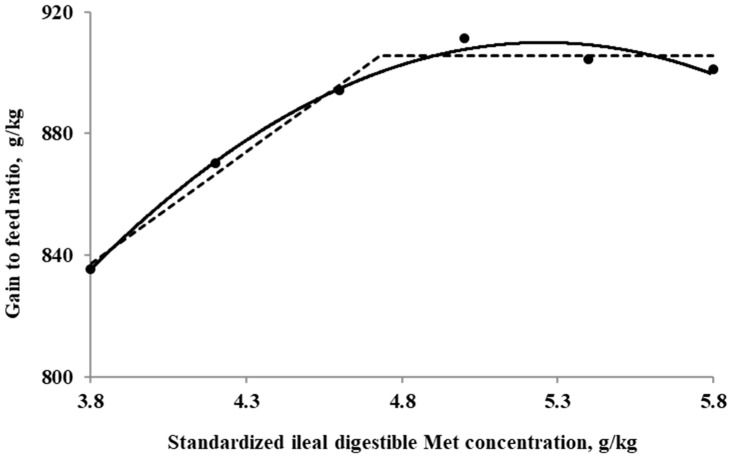
Fitted broken (dotted) and quadratic (solid) lines based on the gain-to-feed ratio (g/kg) in 0–10-day-old broilers as a function of standardized ileal digestible (SID) Met in the diet (Experiment 1). Data points are expressed as the means of eight cages per treatment. The one-slope broken-line model indicates that the SID Met requirement was 4.7 g/kg (Y = 906 + 73.9 × [X − 4.7] [X < 4.7]; *p* = 0.002; R^2^ = 0.983; SE = 0.08; CI 95% = 4.46 to 4.99). The quadratic-line model shows that the SID Met requirement was 5.3 g/kg (Y = 910 − 35.2 × [5.3 − X] × [5.3 − X] [X < 5.3]; *p* < 0.01; R^2^ = 0.991; SE = 0.06; CI 95% = 5.08 to 5.43), and 95% of the upper asymptotic value of the quadratic model indicates that the SID Met requirement was 5.0 g/kg.

**Figure 4:**
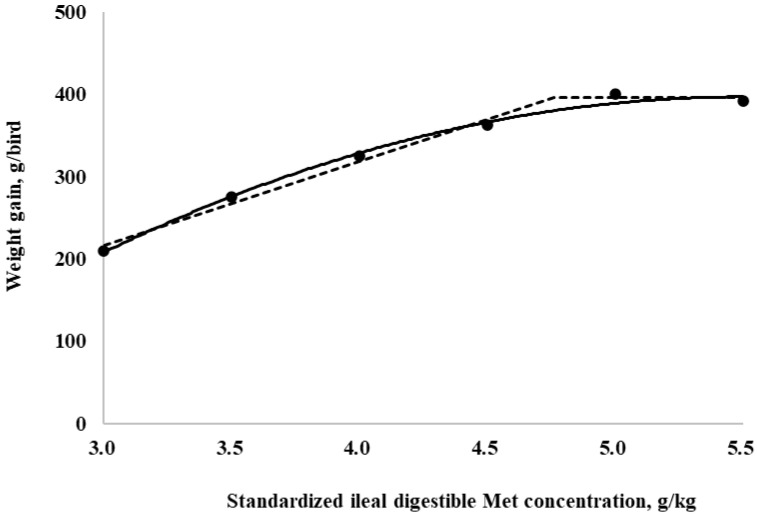
Fitted broken (dotted) and quadratic (solid) lines based on weight gain (g/bird) in 10–21-day-old broilers as a function of standardized ileal digestible (SID) Met in the diet (Experiment 2). Data points are expressed as the means of eight cages per treatment. The one-slope broken-line model indicates that the SID Met requirement was 4.8 g/kg (Y = 396.0 + 101.6 × [X − 4.8] [X < 4.8]; *p* = 0.0008; R^2^ = 0.991; SE = 0.11; CI 95% = 4.4 to 5.1). The quadratic-line model shows that the SID Met requirement was 5.5 g/kg model (Y = 396.9 − 30.0 × [5.5 − X] × [5.5 − X]; *p* < 0.01; R^2^ = 0.993; SE = 0.24; CI 95% = 4.8 to 6.2), and 95% of the upper asymptotic value of the quadratic model indicates that the SID Met requirement was 5.2 g/kg.

**Figure 5:**
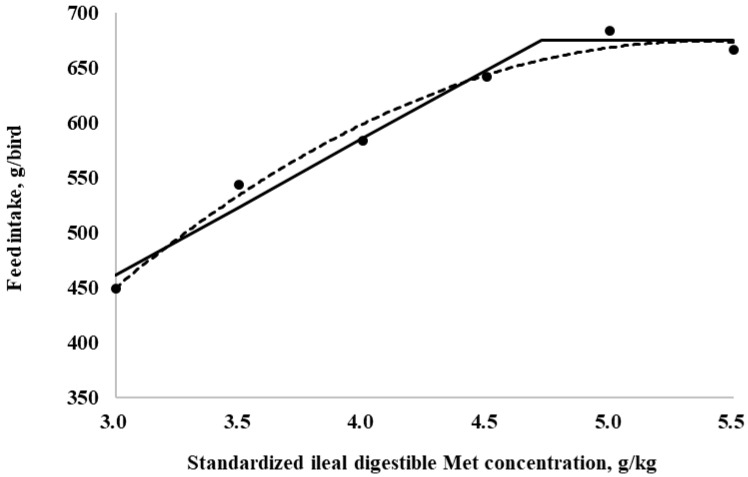
Fitted broken (dotted) and quadratic (solid) lines based on feed intake (g/bird) in 10–21-day-old broilers as a function of standardized ileal digestible (SID) Met in the diet (Experiment 2). Data points are expressed as the means of eight cages per treatment. The one-slope broken-line model indicates that the SID Met requirement was 4.7 g/kg (Y = 674.6 + 123.8 × [X − 4.7] [X < 4.7]; *p* = 0.003; R^2^ = 0.980; SE = 0.16; CI 95% = 4.2 to 5.2). The quadratic-line model shows that the SID Met requirement was 5.4 g/kg model (Y = 673.7 − 39.6 × [5.4 − X] × [5.4 − X]; *p* < 0.01; R^2^ = 0.984; SE = 0.28; CI 95% = 4.5 to 6.3), and 95% of the upper asymptotic value of the quadratic model indicates that the SID Met requirement was 5.1 g/kg.

**Figure 6:**
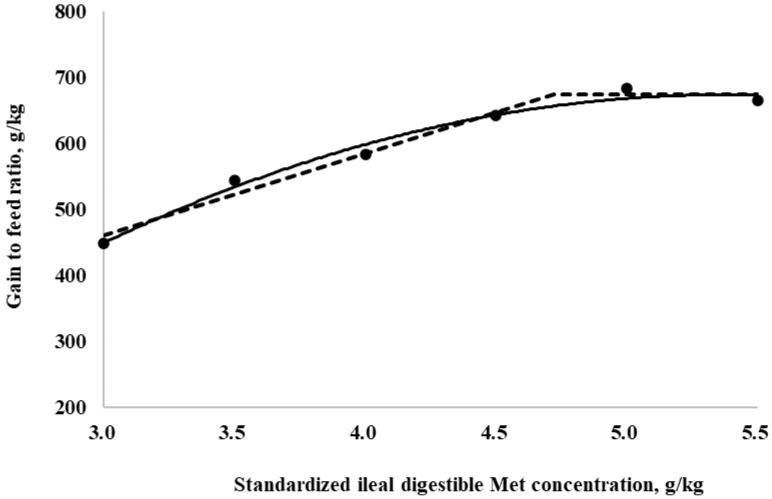
Fitted broken (dotted) and quadratic (solid) lines based on the gain-to-feed ratio (g/kg) in 10–21-day-old broilers as a function of standardized ileal digestible (SID) Met in the diet (Experiment 2). Data points are expressed as the means of eight cages per treatment. The one-slope broken-line model indicates that the SID Met requirement was 4.7 g/kg (Y = 674.6 + 123.8 × [X − 4.7] [X < 4.7]; *p* = 0.003; R^2^ = 0.980; SE = 0.15; CI 95% = 4.2 to 5.2). The quadratic-line model shows that the SID Met requirement was 5.4 g/kg (Y = 673.7 − 39.7 × [5.4 − X] × [5.4 − X] [X < 5.4]; *p* = 0.002; R^2^ = 0.981; SE = 0.03; CI 95% = 4.5 to 6.3), and 95% of the upper asymptotic value of the quadratic model indicates that the SID Met requirement was 5.1 g/kg.

The SID Met requirements for broilers during the starter period (0 to 10 days of age), determined using a linear broken-line model, were 4.4, 4.3, and 4.7 g/kg for BWG, feed intake, and G:F, respectively. Additionally, a quadratic-line model yielded estimated SID Met requirements of 5.1, 5.1, and 5.3 g/kg for BWG, feed intake, and G:F, respectively (Figure 1, Figure 2 and Figure 3). The SID Met requirements for broilers during the grower period (10 to 21 days of age), determined using a linear broken-line model, were 4.8, 4.7, and 4.7 g/kg for BWG, feed intake, and G:F, respectively (Figure 4, Figure 5 and Figure 6). Meanwhile, using a quadratic-line model, the estimated SID Met requirements were 5.5, 5.4, and 5.4 g/kg for BWG, feed intake, and G:F, respectively. Statistically, the quadratic models have relatively high R^2^ values, regardless of the age of the birds (Figure 1, Figure 2, Figure 3, Figure 4, Figure 5 and Figure 6). The quadratic models for 0 to 10 days demonstrated greater reliability with the dataset; however, the results for 10 to 21 days exhibited slightly higher R^2^ values, while the standard errors and confidence limits for the estimated values were even greater compared to the linear models.

## 4. Discussion

Both Met and Cys play essential roles in protein metabolism and cellular activity. Accurate diet formulation requires information on the optimum dietary Met:Cys ratio in TSAA. Several studies have determined the TSAA requirements for broilers [11,12,15,24]. The Met requirement is influenced by the dietary Met concentration [16] and the Met:Cys ratio in TSAA [17,25]. Different recommendations exist for the dietary TSAA in broilers; Rostagno et al. [9] suggest a digestible Met:Cys ratio of 55:44, while the Ross 308 Nutrient Specifications [8] recommend a ratio of 54:46. In addition, an analysis of 15 studies using quadratic models suggested that the recommended SID Met+Cys:Lys ratios were 73% for BWG and 74% for feed conversion ratio (FCR), respectively [14]. The present study indicated SID Met+Cys:Lys ratios of 69% in Experiment 1 (0–10 days old) and 80% in Experiment 2 (10–21 days old). Considering that the conversion of dietary Met to Cys is essential as the demand for Cys increases, it is important to note that feather protein synthesis exhibits a particularly high requirement for Cys. Feathers contain a significantly higher concentration of Cys relative to Met, with a ratio exceeding 10 times (75.3 g/kg of total amino acids for Cys compared to 7.1 g/kg for Met) [26]. Additionally, the covering of feathers and the renewal of feathers in chickens vary according to growth stage [25] and the type of bird, such as those characterized by fast or slow feathering [27].

Previous studies estimating digestible Met requirements formulated experimental diets using two approaches: one based on digestible Met concentrations [28] and the other on TSAA concentrations [11,29]. Two methods for formulating diets to determine dietary AA requirements are available. The first method involves using a basal diet with graded AA levels [30,31], the second method increases the amounts of all dietary AAs, including the test AA, while maintaining the standard ratio [32]. In this study, the latter method was used, where the quantities of corn and SBM were fixed, and synthetic AAs, including Met, were increased equally to achieve the ideal AA ratio. This approach allows for an improved response to Met supplementation when dietary Met is deficient; however, it may lead to excessive supply once required amino acid levels are exceeded. Previous studies have demonstrated that an imbalanced dietary Met:Cys ratio can inhibit growth performance. For example, Wheeler and Latshaw [15] found that increasing Cys supplementation improved BWG and FCR without affecting feed intake. In contrast, Baker [33] reported that excess dietary L-Cys induced acute metabolic acidosis in chickens. In this study, dietary Cys supplementation ranged from 28 to 33 times and 8 to 12 times the recommended levels in Experiments 1 and 2, respectively. However, no toxic or growth depression was observed during the experimental period. This result may be partially explained by Liebig’s bucket principle, which states that the potentially attainable yield depends on the amount of the most limiting nutrient. This result could explain why no toxic effects were observed in the broilers despite excess dietary Cys. To clarify this point, further research is needed to evaluate protein accretion and oxidative stress in response to excessive dietary Met and Cys intake.

Pacheco et al. [25] found that when dietary L-Cys supplementation increased in terms of the Met:Cys ratio (56:44 vs. 44:56), the proportion of Cys originating from dietary Met decreased from 43% to 27%. This result suggests that dietary Cys levels, even within a fixed TSAA range, can influence the Met requirements of birds by modulating the Met cycle. However, Kalinowski et al. [27] observed no growth responses in birds receiving submarginal Met (4.5 g/kg) with increasing dietary Cys levels nor in those receiving adequate Cys with graded Met levels. Because the dietary Cys levels in the present study exceeded the recommended levels but constant ratios to TSAA, the Met requirement estimates obtained from this experiment might not have been influenced by dietary Cys levels.

The variability in reported Met requirements reflects differences in experimental conditions, including definitions of dietary Met concentrations, such as total or digestible basis, statistical models employed, and response criteria. For example, the digestible Met requirements for broilers aged 0 to 21 days observed in 2010 were 4.7 g/kg for BWG and 4.6 g/kg for FCR according to a quadratic-line model [16]. The total Met requirement was reported in 2020 as 5.5 g/kg for average daily gain and 5.8 g/kg for FCR for broilers aged 0 to 21 days [34]. Recently, Macelline et al. [6] reported an average of 3.5 g/kg (3.43 g/kg for BWG and 3.50 g/kg for FCR) of L-methionine supplemented to achieve maximum performance for male broilers aged 1 to 21 days, corresponding to 6.54 g/kg Met in the diet, which exceeds the highest Met concentrations utilized in the experimental diets. Furthermore, the total Met requirement estimate reported nearly 30 years ago was 3.3 g/kg for BWG, using a broken-line model for broilers aged 1 to 21 days [17]. This figure is lower than those found in more recent literature. In addition, the estimated Met requirements for maximum performance in this study (5.3 g/kg for 1 to 10 days and 5.4 g/kg for 10 to 21 days), as well as those reported by commercial sources [7,8,13], exceeded 5.0 g/kg for broilers up to 21 days old. This trend suggests that the recommended levels of Met required to achieve maximum performance are increasing in response to advancements in growth potential resulting from the selective breeding of broilers.

Generally, most of the AA requirements for BWG are lower than those for G:F [30,35], whereas the estimated SID Met requirement of 5.1 g/kg for maximum BWG in the present study was lower than that for G:F (5.3 g/kg) in 0- to 10-day-old birds (Experiment 1). In 10- to 21-day-old birds (Experiment 2), the SID Met requirements (5.5 g/kg for BWG vs. 5.4 g/kg for G:F) exhibited a conflicting pattern compared to previous studies estimating the Lys requirement [30,35]. However, this finding is consistent with results from other investigations of the Met requirements [16,27,28]. Various factors affecting the growth performance of broilers, such as age, environmental conditions (e.g., temperature), diet compositions with different dietary AA ratios, Met:Cys, dietary Lys levels, and genetic background, may contribute to the differences in Met requirement patterns. However, further investigation is needed to fully elucidate the underlying reasons for these discrepancies.

Amino acid requirements can be expressed as a percentage (%) [23,27,28,29,30,31] or in mg/d, or both [36], and are typically reported as a percentage of the diet. Expressing AA requirements as a percentage is advantageous for large-scale feed formulation, as it accurately reflects the proportion of AAs in the feed. Furthermore, expressing AA requirements in milligrams per day (mg/d) provides precise daily intake levels under specific conditions, such as age and feed consumption patterns. According to Aviagen [37] on Ross 308 performance objectives, the predicted feed intake for days 0 to 10 and 10 to 21 is approximately 300 g and 900 g, respectively. In this study, the average feed intake, regardless of dietary SID Met levels, was only 52% and 61% of these predicted values. Body weight corresponding to the birds’ age was delayed by 2 to 4 days compared to Aviagen [37], likely due to external stress from experimental handling and weighing during performance assessments. Despite this delay, feed intake relative to body weight was consistent with data from Aviagen [37], indicating the suitability of the birds for evaluating growth responses under varying SID Met levels.

Broilers generally exhibit a reduction in feed intake when dietary energy density is high, necessitating adjustments to AA concentrations to ensure adequate daily AA intake [38]. NRC [39] reported that 3-week-old male broilers require 0.5% Met in the diet or 217 mg of Met per day. Similarly, the amounts of digestible Met based on data from Aviagen for nutrition specifications [13] and performance objectives [37] for Ross 308 were calculated to be 77.3 mg/d (digestible Met 0.55% for d 0 to 10) and 218 mg (digestible Met 0.51% for d 11 to 24) in birds that cumulatively consumed 877.7 and 2739 kcal metabolizable energy (ME) per bird, respectively. In this study, the estimated Met requirements (mg/bird/day) for optimal and maximal responses were 68.9 and 79.5 (d 0 to 10), and 277 and 343 (d 10 to 21) for weight gain, and 77.5 and 89.8 (d 0 to 10), and 274.5 and 330.3 (d 10 to 21) for G:F. During these periods, the birds consumed diets providing an average of 506.7 and 1784 kcal ME per bird, values lower than those reported in the literature. However, the required Met amounts (mg/d) for supporting certain growth stages of the birds fell within the range of previous reports [37,39]. This suggests that the SID Met levels expressed as percentages, observed in this study, could be utilized for formulating diets for broilers aimed at achieving specific growth performance during the growth stages. 

The estimates obtained from different statistical methods are also used to determine the optimum flock size for the birds. The linear broken-line model suggests that minimum dietary AA levels are necessary for maximum bird growth or productivity [40] or for the required values for the average birds in the population [41]. In contrast, the quadratic model represents the recommended nutrients for maximum growth in birds corresponding to 100% of the population. However, there are various ways to identify reliable regression models. For example, selecting models with high R^2^ values and low standard errors can lead to narrower confidence intervals for the estimates, indicating greater precision. Alternatively, choosing the model with the smallest AICc or evaluating models based on relative likelihood (e.g., evidence ratios) can provide a robust foundation for determining which model best fits the data [42]. By considering these metrics (e.g., R^2^ and standard errors) in conjunction with the specific objectives for the bird population, a more comprehensive interpretation of nutrient requirement estimates can be achieved. Although these estimates may not be fully acceptable as definitive Met recommendations for achieving specific growth responses in broilers, they do provide an example of the levels required to attain optimal and maximal performance in the particular population used in this study. Therefore, further investigations are needed to confirm these recommended levels under commercial conditions.

## 5. Conclusions

In conclusion, the estimated Met requirements for optimal BWG were 4.4 g/kg (68.9 mg/d) and 4.8 g/kg (277.1 mg/d) in the starter (0 to 10 days of age) and grower (10 to 21 days of age) phases, respectively. The G:F indicated a Met requirement of 4.7 g/kg (77.5 mg/d for starter; 274.5 mg/d for grower) for both phases. Furthermore, for maximum BWG, the estimated SID Met requirements were 5.1 g/kg (79.5 mg/d) and 5.5 g/kg (343.0 mg/d) in the starter and grower phases, respectively. The G:F indicated Met requirements of 5.3 g/kg (89.8 mg/d) and 5.4 g/kg (330.3 mg/d) in the respective phases. The varying Met requirements derived from different statistical approaches will provide valuable insights for nutritionists involved in formulating broiler diets, particularly when addressing specific demand considerations. To enhance our understanding of Met requirements for broilers, further research is needed to assess how the recommended levels of Met may fluctuate under different dietary Met-to-Cys ratios or varying environmental temperatures.

## Figures and Tables

**Table 1:** Ingredients and chemical compositions (g/kg) of the experimental diets for broilers aged 1–10 days (Experiment 1; as-fed basis).

Ingredient, g/kg	Standardized Ileal Digestible Met Concentration, g/kg					
	3.8	4.2	4.6	5.0	5.4	5.8
Corn	320.0	320.0	320.0	320.0	320.0	320.0
Soybean meal	235.0	235.0	235.0	235.0	235.0	235.0
Corn starch	256.5	262.8	269.0	275.2	281.5	287.6
Glutamic acid	81.9	65.5	49.1	32.7	16.3	-
Soybean oil	20.0	20.0	20.0	20.0	20.0	20.0
L-Lysine-HCl	8.8	10.6	12.4	14.2	16.0	17.9
DL-Methionine	2.1	2.5	2.9	3.3	3.7	4.1
L-Threonine	3.8	4.6	5.5	6.3	7.2	8.1
L-Tryptophan	0.3	0.5	0.7	0.9	1.1	1.3
L-Arginine	5.4	6.8	8.3	9.8	11.2	12.6
L-Cysteine	3.9	4.5	5.1	5.7	6.2	6.7
L-Histidine	0.9	1.3	1.8	2.2	2.7	3.1
L-Isoleucine	4.2	5.1	6.0	7.1	8.1	9.0
L-Leucine	4.5	6.1	7.6	9.1	10.6	12.2
L-Phenylalanine	1.6	2.4	3.2	4.0	4.8	5.6
L-Valine	5.3	6.5	7.6	8.7	9.8	11.0
Limestone	13.0	13.0	13.0	13.0	13.0	13.0
Dicalcium phosphate	19.8	19.8	19.8	19.8	19.8	19.8
Salt	3.0	3.0	3.0	3.0	3.0	3.0
Sodium carbonate	4.0	4.0	4.0	4.0	4.0	4.0
Vitamin premix ^1^	2.0	2.0	2.0	2.0	2.0	2.0
Mineral premix ^2^	2.0	2.0	2.0	2.0	2.0	2.0
Choline chloride	2.0	2.0	2.0	2.0	2.0	2.0
Total	1000.0	1000.0	1000.0	1000.0	1000.0	1000.0
Calculated values, g/kg						
Metabolizable energy, kcal/kg	3257	3278	3299	3320	3341	3362
Crude protein	225	225	225	225	225	225
Calcium	10.0	10.0	10.0	10.0	10.0	10.0
Non-phytate P	4.5	4.5	4.5	4.5	4.5	4.5

^1^ Supplies the following per kilogram of diet: vitamin A, 24,000 IU; vitamin D_3_, 8000 IU; vitamin E, 160 mg/kg; vitamin K_3_, 8 mg/kg; vitamin B_1_, 8 mg/kg; vitamin B_2_, 20 mg/kg; vitamin B_6_, 12 mg/kg; pantothenic acid, 40 mg/kg; folic acid, 4 mg/kg; niacin, 12 mg/kg. ^2^ Supplies the following per kilogram of diet: Fe, 120 mg/kg; Cu, 320 mg/kg; Zn, 200 mg/kg; Mn, 240 mg/kg; Co, 2 mg/kg; Se, 0.6 mg/kg; I, 2.5 mg/kg.

**Table 2:** Calculated standardized ileal digestible indispensable amino acid composition of the experimental diets containing six graded levels of standardized ileal digestible methionine (Experiment 1; as-fed basis).

Item, g/kg	Standardized Ileal Digestible Met Concentration, g/kg					
	3.8	4.2	4.6	5.0	5.4	5.8
SID amino acids						
Arginine	13.75	15.20	16.64	18.09	19.54	20.99
Histidine	4.08	4.50	4.93	5.36	5.79	6.22
Isoleucine	9.17	10.13	11.10	12.06	13.03	13.99
Leucine	14.42	15.94	17.46	18.98	20.50	22.02
Lysine	13.48	14.90	16.32	17.74	19.16	20.58
Methionine	3.80	4.20	4.60	5.00	5.40	5.80
Cysteine	5.54	6.12	6.70	7.29	7.87	8.45
Phenylalanine	7.52	8.32	9.11	9.90	10.69	11.48
Threonine	8.05	8.90	9.74	10.59	11.44	12.28
Tryptophan	1.78	1.97	2.15	2.34	2.53	2.72
Valine	10.65	11.77	12.89	14.01	15.13	16.25

SID = standardized ileal digestible.

**Table 3:** Ingredients and chemical compositions (g/kg) of the experimental diets for broilers aged 10–21 days (Experiment 2; as-fed basis).

Ingredient, g/kg	Standardized Ileal Digestible Met Concentration, g/kg					
	3.0	3.5	4.0	4.5	5.0	5.5
Corn	350.0	350.0	350.0	350.0	350.0	350.0
Soybean meal	200.0	200.0	200.0	200.0	200.0	200.0
Corn starch	242.4	247.8	253.5	259.2	265.0	270.8
Glutamic acid	121.1	108.7	94.0	78.9	63.8	48.7
Soybean oil	25.0	25.0	25.0	25.0	25.0	25.0
L-Arginine	-	1.5	2.8	4.2	5.5	6.8
L-Histidine	-	0.1	0.6	1.0	1.4	1.9
L-Isoleucine	0.5	1.4	2.3	3.2	4.1	5.0
L-Leucine	-	-	1.2	2.6	3.9	5.3
L-Lysine-HCl	2.0	3.6	5.2	6.8	8.4	9.9
DL-Methionine	1.3	1.8	2.3	2.8	3.3	3.8
L-Cysteine	1.4	1.9	2.4	2.9	3.4	3.9
L-Phenylalanine	-	-	0.5	1.2	2.0	2.7
L-Threonine	0.9	1.7	2.5	3.3	4.1	4.9
L-Tryptophan	-	0.1	0.3	0.5	0.7	0.9
L-Valine	1.0	2.0	3.0	4.0	5.0	6.0
Limestone	17.1	17.1	17.1	17.1	17.1	17.1
Dicalcium phosphate	18.0	18.0	18.0	18.0	18.0	18.0
Salt	4.5	4.5	4.5	4.5	4.5	4.5
Sodium carbonate	4.0	4.0	4.0	4.0	4.0	4.0
Vitamin premix ^1^	5.0	5.0	5.0	5.0	5.0	5.0
Mineral premix ^2^	5.0	5.0	5.0	5.0	5.0	5.0
Choline chloride	0.8	0.8	0.8	0.8	0.8	0.8
Total	1000.0	1000.0	1000.0	1000.0	1000.0	1000.0
Calculated values, g/kg						
Metabolizable energy, kcal/kg	3231	3243	3261	3281	3300	3319
Crude protein	200	200	200	200	200	200
Calcium	10.0	10.0	10.0	10.0	10.0	10.0
Non-phytate P	4.5	4.5	4.5	4.5	4.5	4.5

^1^ Supplies the following per kilogram of diet: vitamin A, 24,000 IU; vitamin D_3_, 8000 IU; vitamin E, 160 mg/kg; vitamin K_3_, 8 mg/kg; vitamin B_1_, 8 mg/kg; vitamin B_2_, 20 mg/kg; vitamin B_6_, 12 mg/kg; pantothenic acid, 40 mg/kg; folic acid, 4 mg/kg; niacin, 12 mg/kg. ^2^ Supplies the following per kilogram of diet: Fe, 120 mg/kg; Cu, 320 mg/kg; Zn, 200 mg/kg; Mn, 240 mg/kg; Co, 2 mg/kg; Se, 0.6 mg/kg; I, 2.5 mg/kg.

**Table 4:** Calculated standardized ileal digestible indispensable amino acid composition of the experimental diets containing six graded levels of standardized ileal digestible methionine (Experiment 2; as-fed basis).

Item, g/kg	Standardized Ileal Digestible Met Concentration, g/kg					
	3.0	3.5	4.0	4.5	5.0	5.5
SID amino acids						
Arginine	7.70	9.19	10.50	11.81	13.13	14.44
Histidine	2.95	3.06	3.50	3.94	4.38	4.81
Isoleucine	5.25	6.13	7.00	7.88	8.75	9.63
Leucine	9.50	9.50	10.70	12.04	13.38	14.71
Lysine	7.50	8.75	10.00	11.25	12.50	13.75
Methionine	3.00	3.50	4.00	4.50	5.00	5.50
Cysteine	3.00	3.50	4.00	4.50	5.00	5.50
Phenylalanine	5.54	5.54	6.00	6.75	7.50	8.25
Threonine	4.88	5.69	6.50	7.31	8.13	8.94
Tryptophan	1.31	1.40	1.60	1.80	2.00	2.20
Valine	6.00	7.00	8.00	9.00	10.00	11.00

SID = standardized ileal digestible.

**Table 5:** Growth performance of male broilers aged 0 to 10 days fed diets containing different dietary standardized ileal digestible methionine concentrations in Experiment 1 ^1,4^.

Item ^2^	Dietary SID Methionine Concentration, g/kg ^2^						SEM ^3^	*p*-Values	
	3.8	4.2	4.6	5.0	5.4	5.8		Linear	Quadratic
Initial BW, g	39.4	39.4	39.4	39.5	39.4	39.4	1.00	0.967	0.989
Final BW, g	144.5	169.3	180.4	184.9	178.3	174.8	3.29	<0.01	<0.01
Weight gain, g/bird	105.1	129.9	140.9	145.5	138.9	135.4	2.64	<0.01	<0.01
Feed intake, g/bird	126.1	149.4	158.2	160.3	154.7	151.9	4.89	0.001)	<0.01
G:F, g/kg	835.3	870.2	894.4	911.3	904.5	901.1	25.22	0.039	0.179

^1^ Values are least-squares means of eight replicate cages containing 15 birds each. ^2^ BW = body weight; G:F = gain-to-feed ratio; SID = standardized ileal digestible. ^3^ SEM = standard error of the mean. ^4^ The AICc values for weight gain, feed intake, and gain-to-feed ratio from linear and quadratic models were 373 and 337, 373 and 347, and 435 and 408, respectively.

**Table 6:** Growth performance of male broilers aged 10 to 21 days fed diets containing different dietary standardized ileal digestible methionine concentrations in Experiment 2 ^1,4^.

Item ^2^	Dietary SID Methionine Concentration, g/kg ^2^						SEM ^3^	*p*-Values	
	3.0	3.5	4.0	4.5	5.0	5.5		Linear	Quadratic
Initial BW, g	313	307	309	311	311	309	5.9	0.94	0.85
Final BW, g	527	587	637	673	712	702	14.0	<0.01	0.004
Weight gain, g/bird	210	276	325	363	400	392	11.1	<0.01	0.0002
Feed intake, g/bird	468	506	557	564	586	589	14.1	<0.01	0.034
G:F, g/kg	449	544	583	642	684	666	12.4	<0.01	<0.01

^1^ Values are least-squares means of eight replicate cages containing six birds each. ^2^ BW = body weight; G:F = gain-to-feed ratio; SID = standardized ileal digestible. ^3^ SEM = standard error of the mean. ^4^ The AICc values for weight gain, feed intake, and gain-to-feed ratio from linear and quadratic models were 470 and 449, 481 and 470, and −149 and −160, respectively.

## Data Availability

The original contributions presented in the study are included in the article, further inquiries can be directed to the corresponding author.
